# The Utility of Gait Deviation Index (GDI) and Gait Variability Index (GVI) in Detecting Gait Changes in Spastic Hemiplegic Cerebral Palsy Children Using Ankle–Foot Orthoses (AFO)

**DOI:** 10.3390/children7100149

**Published:** 2020-09-25

**Authors:** Majewska Joanna, Szczepanik Magdalena, Bazarnik-Mucha Katarzyna, Szymczyk Daniel, Lenart-Domka Ewa

**Affiliations:** Institute of Health Sciences, Medical College, University of Rzeszow, 35-959 Rzeszów, Poland; joadud@gmail.com (M.J.); szczepanikmp@gmail.com (S.M.); daniel.szymczyk@op.pl (S.D.); e.domka@op.pl (L.-D.E.)

**Keywords:** gait analysis, cerebral palsy, gait variablity index, gait deviation index, ankle–foot orthosis

## Abstract

Background: Cerebral palsy (CP) children present complex and heterogeneous motor disorders that cause gait deviations. Clinical gait analysis (CGA) is used to identify, understand and support the management of gait deviations in CP. Children with CP often use ankle–foot orthosis (AFO) to facilitate and optimize their walking ability. The aim of this study was to assess whether the gait deviation index (GDI) and the gait variability index (GVI) results can reflect the changes of spatio-temporal and kinematic gait parameters in spastic hemiplegic CP children wearing AFO. Method: The study group consisted of 37 CP children with hemiparesis. All had undergone a comprehensive, instrumented gait analysis while walking, both barefoot and with their AFO, during the same CGA session. Kinematic and spatio-temporal data were collected and GVI and GDI gait indexes were calculated. Results: Significant differences were found between the barefoot condition and the AFO conditions for selected spatio-temporal and kinematic gait parameters. Changes in GVI and GDI were also statistically significant. Conclusions: The use of AFO in hemiplegic CP children caused a statistically significant improvement in spatio-temporal and kinematic gait parameters. It was found that these changes were also reflected by GVI and GDI. These findings might suggest that gait indices, such as GDI and GVI, as clinical outcome measures, may reflect the effects of specific therapeutic interventions in CP children.

## 1. Introduction

Cerebral palsy (CP) with a prevalence of about 2.2/1000 live births is the most common cause of motor disability in childhood [1]. Changes in gait of children with spastic cerebral palsy are often affected by symptoms of spasticity and lower extremity muscle weakness, which limit the patient’s ability to walk [2].

The use of ankle–foot orthoses (AFOs) is widely recommended to prevent the development or progression of deformity and to improve dynamic efficiency of the child’s gait [3]. There are a wide variety of AFOs used in clinical practice, which are differentiated depending on their design, the material used and the stiffness of that material. Changing any of these three elements will alter the AFO control and this may have influence on the patient’s gait [4]. Because of heterogeneity among study designs, AFO types, control conditions, and outcome measures, main effects of AFO on gait of children with CP are unclear. Furthermore, most studies in this area are focused on evaluating a single, specific outcome measure (e.g., step length, ankle range of motion, or knee flexion) rather than the overall effect of AFO use on patient’s gait [5]. 

Three-dimensional gait analysis (3DGA) is commonly used in clinical practice and scientific research for the purpose of objective assessment and description of gait disorders, as well as to plan and evaluate the treatment of children with CP. This method provides a large amount of interdependent data and variables concerning spatio-temporal gait parameters, joint motions (kinematics), as well as joint movement and power (kinetics) in three planes [6]. Given the different gait patterns and pathologies in children with CP, a global analysis is essential in clinical practice. For this purpose, it is useful to summarize and present the results from 3DGA as a single, quantitative, numerical measure that reflects the patient’s gait. 

One of the models designed to obtain a single measure of the quality of a gait pattern is the gait deviation index (GDI), that measures the subject’s gait deviation from a reference, normative data. It is a score derived from 3DGA, which provides a numerical value that expresses overall gait pathology (range 0–100, where 100 and above indicates absence of gait pathology). Every 10-point decrease in the GDI corresponds to one standard deviation from the mean of healthy controls. GDI is calculated based on kinematic parameters [7]. 

Another gait index, the gait variability index (GVI), is computed based on spatio-temporal parameters. The GVI was intended to be applicable to different patient’s groups and diseases severities. It was constructed as a complex measure of nine weighted spatio-temporal gait variables seen in relation to a reference population. GVI value ≥ 100 indicates a similar level of gait variability as the reference population, and each 10-point reduction in the score corresponds to one standard deviation from the reference mean, where lower scores indicate increased gait variability [8,9].

Danino et al. have demonstrated that improvement in temporal and kinematic parameters in spastic diplegic CP children using AFO are not reflected by some gait indices, including GDI [10]. Therefore, the aim of this study was to assess whether the GDI and the GVI reflect changes in temporal and kinematic parameters in spastic hemiplegic CP children wearing AFO.

## 2. Materials and Methods

### 2.1. Participants

The study was approved by the Bioethics Committee of the Faculty of Medicine of the University of Rzeszow, Poland (9/2/2017). All children and their parents were informed about the procedures of this study and have signed written informed consent. All measurements were performed in accordance to the Declaration of Helsinki. Participants were recruited from the rehabilitation center for children and youth in Poland. The study population included 37 pediatric patients (19 male and 18 female, with a mean age of 13 years and 7 months). Sixteen children had a right hemiparetic CP and 21 had a left hemiparetic CP (Table 1). All participants walked without additional aids.

Inclusion criteria for hemiplegic CP children were: unilateral, spastic CP as defined by Bax et al. [11]; ability to walk independently at least 10 m; and age between 6 and 18 years. Exclusion criteria were: a previous orthopedic or neurosurgical intervention; botulinum toxin injections within the last 12 months; systemic, anti-spasticity medications; and inability to understand the oral instructions given during the gait analysis. All participants had the posterior leaf spring AFO (PLS) prescribed by their physician. It allows slight plantarflexion, as well as dorsiflexion in stance to promote ‘normal’ ankle rocker function and to create more dynamic gait. All children had been wearing the PLS for at least 1 month before the gait analysis. 

### 2.2. Data Collection

3D-gait analysis was performed using a six-camera (120 Hz) movement analysis system with passive markers (BTS Smart, Milan, Italy).

The study procedure included:

− calibration of the system
− anthropometric measurements (body height and weight, lower limbs length, pelvis width and depth of the pelvis, knee joint and the ankle joint width)
− application of the markers according to the modified Davis Protocol
− data recording
− data elaboration and processing using BTS tracker and BTS analyzer software (BTS Bioengineering, Italy).

Reflective markers were placed at defined anatomical points on the pelvis and lower limbs, according to the modified Davis protocol [12]. When walking with the posterior leaf spring AFO and shoes, the lateral malleolus markers were placed on the skin, and the heel and toe markers were placed on the shoes at the positions best projecting the anatomical landmarks (at the level of calcaneous bone tuberosity aligned with the marker at the level of fifth metatarsal). All other markers remained at the same positions throughout the testing procedure. All subjects were asked to walk along a 12-m walkway at self-selected speed, both barefoot and wearing their AFO, in the random order. For each condition, six successive gait trials were recorded and the results of an average of six full gait cycles were subject for further analysis. The same experienced specialist performed these 3DGAs. 

Selected spatio-temporal parameters of the gait (the stance phase—percentage of the gait cycle, step length, gait speed, and gait cadence), as well as selected, specific kinematic parameters of the gait (ankle dorsiflexion at initial contact, maximal ankle dorsiflexion at swing, knee flexion at initial contact, maximal knee flexion at swing, knee + hip flexion), for both affected and non-affected lower limb, were computed and analyzed.

### 2.3. GDI Calculations

The GDI was determined based on the following 9 kinematic parameters: pelvic and hip range of motion in all three planes, knee flexion and extension, ankle dorsiflexion and plantarflexion, and foot progression. GDI values were calculated using the Excel spreadsheet developed by Schwartz and Rozumalski [7].

### 2.4. GVI Calculations

The GVI was determined based on the following nine spatiotemporal gait parameters: step length (cm), stride length (cm), step time (s), stride time (s), swing time (s), single support time (s), double support time (s), velocity (cm/s), and standard deviations (SDs) of each parameter. GVI values were computed using the Excel spreadsheet developed by Gouelle et al. [9].

### 2.5. Statistical Analysis

The significance of the differences of the gait parameters in the study group between the two conditions (walking with orthoses and walking barefoot) was calculated using a non-parametric Wilcoxon test. The significance of differences between these two conditions was assessed using a non-parametric, precise version of this test for small samples. The level of significance was assumed at *p* < 0.05. Statistical analyses were performed using Statistica software (StatSoft, ver. 10.0, Krakow, Poland).

## 3. Results

### 3.1. Spatio-Temporal Gait Parameters

There were no significant differences between the percentage of the stance phase in the gait cycle (neither for the paretic limb nor for the non-paretic limb), comparing walking with orthoses and without them. On the other hand, statistically significant differences were found in the relative step length for both lower limbs. Significantly worse results are obtained when walking barefoot. Similar relationships were observed in the gait speed. The subjects walked with significantly lower gait speed, when walking barefoot. Additionally, gait cadence in the hemiplegic children group, when using AFO, was significantly reduced (*p* = 0.001) (Table 2).

### 3.2. Kinematic Parameters

Using AFO cause a statistically significant increase in ankle dorsiflexion at initial contact compared to walking barefoot on both, the affected (*p* = 0.001) and non-affected side (*p* = 0.019). Knee flexion at initial contact, when using AFOs, was reduced by 7.6° (*p* = 0.038) on the affected side, while no significant reduction of the knee flexion at initial contact on the non-affected side was observed (*p* = 0.09). 

To assess the effect of AFOs on the swing phase, we looked at the maximal ankle dorsiflexion during swing phase and maximal knee flexion in swing phase. Ankle dorsiflexion was significantly increased, when using AFOs, on both sides (*p* = 0.002 and 0.041). Knee flexion was not significantly reduced by the use of AFOs.

To evaluate foot clearance, we calculated the combined maximal flexion of the two joints. Combined flexion at the knee and hip joints showed a significant reduction, for both, the affected and non-affected lower limbs (*p* = 0.031 and 0.046) (Table 3).

### 3.3. Gait Deviation Index

When using AFO, the GDI values increased from 68.6 to 75.1 (*p* = 0.029) in the affected lower limb and from 77.9 to 82.3 (*p* = 0.047) in the non-affected lower limb, which was a statistically significant improvement (Table 4).

### 3.4. Gait Variability Index

The GVI values also increased from 74.2 to 83.1 (*p* < 0.001) and from 78.6 to 86.5 (*p* < 0.001) in the affected and non-affected lower limbs, respectively, which was a statistically significant improvement (Table 5).

## 4. Discussion

Gait impairments are common in children and adolescents with CP. In spastic hemiplegic CP, decreased walking speed, increased stance phase on non-affected leg, as well as longer gait stride are observed. The stride is usually shorter on affected leg and cadence is increased to maintain gait speed [13]. Gait pattern can be impaired due to motor coordination, balance, and stability problems during walking. Additionally, there are significant differences in kinematic parameters of the hip, knee, and ankle joints, compared to healthy children [14]. For the purpose of the improvement of the dynamic gait efficiency, different types of AFO are recommended for patients with spastic hemiplegic CP [15]. 

Step length and gait speed are commonly used to assess patient’s gait quality [16]. Hayek et al. [17] compared gait of the children with CP, while walking barefoot and with AFOs. In hemiplegic group, stride length increased by 11.7% with AFOs, in both affected (10.2%) and non-affected (12.4%) lower leg. The gait cadence decreased by 9.7%, but walking speed was not improved. Additionally, the authors of this study assessed the impact of using AFOs on the kinematic gait parameters. The ankle dorsiflexion at initial contact increased by 9.4° on the affected side and by 5.87° on the non-affected side, on average. The ankle dorsiflexion at swing was improved about 9.9°, while the knee flexion at initial contact on the affected side was decreased by 8.5° [17]. In their study, Wren et al. compared the gait of children with CP (five patients with spastic diplegia and five with spastic hemiplegia), while walking barefoot and with two types of orthotic devices (dynamic ankle–foot orthosis—DAFO and adjustable dynamic response—ADR AFO). In both types of orthoses children improved stride length, hip extension, and dorsiflexion in swing phase. Using of ADR-AFOs produced better push-off power and knee extension, but the level of activity and patient’s satisfaction were higher for DAFOs [18]. Liu et al. evaluated the changes of foot and ankle motion in long follow-up studies (18 months) in the group of 23 children with spastic CP (7 hemiplegic, 16 diplegic), during which patients were using solid ankle–foot orthoses (SAFOs), hinged ankle–foot orthoses (HAFOs), or supramalleolar orthoses (SMO). They observed that the long term use of AFOs can lead to maintaining or improving foot and ankle motion and function [15]. In our study, the step length was longer while the subjects were walking with AFO, comparing to walking barefoot, the gait speed has also improved significantly and the gait cadence was reduced. We have also observed improvement of kinematic parameters, including an increase of the ankle dorsiflexion in stance and in swing phase in both legs. Moreover, the knee flexion at the initial contact decreased significantly only in the affected leg. We did not observe significant changes at the knee flexion during the swing phase, but, both hip and knee flexion range of motion were improved.

In our study, we also evaluated whether the GDI and the GVI can reflect changes in spatio-temporal and kinematic parameters in spastic hemiplegic CP children wearing AFO. GDI and GVI indexes were developed to present how far an individual’s gait differs from a normal gait pattern [7,9]. The GDI has been used to assess the gait of children with CP [5,10,16,19,20]. However, we did not find any scientific researches concerning using GVI in this group of patients. This fact encouraged us to check if GVI will be useful to assess changes in the gait of children with hemiplegic CP.

In our study, as it was expected, the value of both gait indices increased, along with the improvement of the selected spatio-temporal and kinematic gait parameters, when the children were wearing AFO. Bickley et al. evaluated two outcome measures: technical surgical goals system (TAG) and GDI and how they reflect changes in postoperative status in children with spastic CP. The TAGs goals have been developed at Shriners Hospital for Children in Huston in 1994 to reflect expected clinical examination and kinematic changes after the surgical treatment. In their study, they observed that using both GDI and TAGs system can improve postoperative assessment in patients with CP at different level of Gross Motor Function Classification System (GMFCS) (I–III). They did not observe any significant differences concerning changes of the GDI results after surgical treatment between different levels (I–III) of GMFCS [19]. Reis et al. showed, that wearing of AFO significantly improves step length, gait speed, and GDI in children with diplegic CP, but only changes in step length was clinically important [16]. A few studies show correlation between GDI and GMFCS. Malt et al. concluded that GDI can be useful to evaluate and present walking impairments in children with spastic CP [6]. Molloy et al. suggest that gait problems may be not well enough recognized when only GMFCS is used; while GDI, as a specific tool for gait assessment, can better reflect functional gait aspects and components [20].

On the other hand, Massaad et al. pointed out that the main clinical utility of GDI is the assessment of the global changes in patient’s gait after the intervention, expressed as a single, numerical value, but without specific information about the source or nature of these observed changes [5]. Additionally, Domino et al. showed that the improvement in spatio-temporal and kinematic parameters of gait in children with diplegic CP did not correspond with changes in GDI, Gillette gait index (GGI), or gait profile score (GPS) [10]. In contrast to our study, which has shown that the changes in spatio-temporal parameters and gait kinematics were reflected in GDI and GVI results changes. Galli et al. evaluated the gait of hemiplegic and diplegic children with CP using other gait indices: GPS and gait variable score (GVS), which are also based on kinematic gait parameters. The children were assessed barefoot and while wearing AFO. They concluded that the gait indexes can be useful in evaluation of immediate effects of using the AFOs in hemiplegic and diplegic CP patients, but it was observed only in some GVS parameters—pelvic tilt and ankle dorsiflexion—but not in GPS [21].

The GVI, based on nine spatiotemporal gait parameters was developed and by Gouelle et al. in 31 patients with Friedreich’s Ataxia. They showed significant decrease of GVI in patients with Friedreich’s Ataxia (70.4 ± 7.9) compared to the healthy subjects (100.3 ± 8.6) [9]. Guzik et al. used GVI for the gait assessment in patients after ischemic stroke. In the control group of the healthy-matched subjects, the GVI scores were 98.34 ± 6.83 for the right lower limb and 96.3 ± 7.19 for the left lower limb and there were significantly different, comparing to the patients after stroke (76.32 ± 7.98 affected leg, 80.74 ± 4.68 non-affected leg) [22]. In the validation study of GVI in patients after stroke in a chronic stage of recovery, the same authors showed that the GVI for the affected and unaffected leg were significantly correlated with the results of clinical functional assessment [23]. Balasubramanian et al. evaluated changes in spatio-temporal gait parameters in two groups of old people, younger and older adults (age < 65 and age ≥ 65, respectively) with GVI. They observed significantly reduced GVI in older adults (91.92 ± 8.75) compared to the younger adults (100.79 ± 7.99). Additionally, they pointed out that low level of functional mobility was correlated with lower value of GVI [24]. GVI score was also validated for patients with mild to moderate Parkinson’s disease. The results showed that a mean, overall value of GVI was 97.5 ± 11.7 and mean GVI value for the more affected side was 94.5 ± 10.6 [8]. In our study GVI value while walking barefoot was 74.2 ± 9.48 for affected leg and 78.6 ± 7.67 for non-affected leg and increased significantly when the subjects were wearing AFO (83.1 ± 8.74 for affected leg, 86.5 ± 8.32 for non-affected leg).

### Study Limitation

One of the major limitations is the fact that only children at GMFCS level I were included in our study group. Further studies will be necessary, including patients other than GMFCS level I, as well as investigating relationships between the GMFCS level of the children and the quantitative gait assessment, including various gait indices and gait symmetry indices. 

## 5. Conclusions

The improvement of the selected spatio-temporal and kinematic gait parameters, when using AFO, is also reflected in the results of GDI and GVI. These gait indices, being the single, numerical parameter, reflecting changes in gait pattern after various therapeutical interventions, would be also valuable in the clinical practice in the group of spastic hemiplegic CP children.

## Figures and Tables

**Table 1:** Characteristics of the study population

	Number (%)	Mean
Gender		
Male	19 (51.4)	
Female	18 (48.6)	
Age (years)		13.7 ± 4.2
Weight (kg)		47.3 ± 9.1
Height (cm)		155.07 ± 11.5
BMI (kg/m^2^)		19.5 ± 2.4
Affected side		
Right	16 (43.2)	
Left	21 (56.7)	
GMFCS I	37 (100)	

BMI—Body Mass Index, GMFCS—Gross Motor Function Classification System.

**Table 2:** Spatio-temporal gait parameters

Parameters	Mean	SD	*p*
Stance phase affected leg BF (% of gait cycle)	60.1	3.3	0.127
Stance phase affected leg AFO (% of gait cycle)	59.3	3.3	
Stance phase non-affected leg BF (% of gait cycle)	62.7	4.8	0.764
Stance phase non-affected leg AFO (% of gait cycle)	62.5	4.1	
Step length affected leg BF (cm)	0.60	0.12	0.001
Step length affected leg AFO (cm)	0.66	0.11	
Step length non-affected leg BF (cm)	0.64	0.11	0.002
Step length no -affected leg AFO (cm)	0.69	0.12	
Gait speed BF (m/s)	0.96	0.22	0.002
Gait speed AFO (m/s)	1.11	0.23	
Cadence (step/min) BF	128.7	21.3	0.001
Cadence (step/min) AFO	118.23	21.3	

BF—barefoot, AFO—ankle–foot orthosis, SD- standard deviation, *p* - significance level

**Table 3:** Kinematic parameters

Parameters	BF		AFO		*p*
	Mean	SD	Mean	SD	
Affected leg ankle dorsiflexion at initial contact (°)	−4.73	5.31	4.34	4.26	0.001
Non-affected leg ankle dorsiflexion at initial contact (°)	2.33	5.42	8.27	7.92	0.019
Affected leg max. ankle dorsiflexion at swing (°)	−2.03	7.43	8.29	6.62	0002
Non-affected leg max. ankle dorsiflexion at swing (°)	6.21	7.24	10.32	7.13	0.041
Affected leg knee flexion at initial contact (°)	27.13	9.47	19.48	9.31	0.038
Non-affected leg knee flexion at initial contact (°)	21.42	8.91	17.06	8.67	0.09
Affected leg max. knee flexion at swing (°)	67.04	9.58	64.66	9.41	0.059
Non-affected leg max. knee flexion at swing (°)	65.88	19.58	68.75	7.29	0.106
Affected leg knee + hip flexion (°)	121.21	11.1	114.37	10.82	0.031
Non-affected leg knee + hip flexion (°)	117.19	12.71	112.02	9.23	0.046

BF—barefoot, AF—ankle–foot orthosis.

**Table 4:** Changes in GDI

	Mean	SD	*p*-Value
GDI affected leg BF	68.6	12.3	0.029
GDI affected leg AFO	75.1	10.7	
GDI non-affected leg BF	77.9	10.4	0.047
GDI non-affected leg AFO	82.3	9.7	

BF—barefoot, AFO—ankle–foot orthosis, GDI—gait deviation index.

**Table 5:** Changes in GVI

	Mean	SD	*p*-Value
GVI affected leg BF	74.2	9.48	<0.001
GVI affected leg AFO	83.1	8.74	
GVI non-affected leg BF	78.6	7.67	<0.001
GVI non-affected leg AFO	86.5	8.32	

BF—barefoot, AFO—ankle–foot orthosis, GVI—gait variability index.
